# Exophytic Nodule on the Finger

**DOI:** 10.7759/cureus.19137

**Published:** 2021-10-29

**Authors:** Renat R Ahatov, Paige Hoyer, MD, Elise Weisert, Brent Kelly

**Affiliations:** 1 Dermatology, University of Texas Medical Branch, Galveston, USA

**Keywords:** myopericytoma on the finger, spindle cell, benign tumor, exophytic nodule, myopericytoma

## Abstract

Myopericytoma is a rare, benign growth characterized by painless lesions with a predilection for the extremities, although they may be found in or on any part of the body. These tumors typically present as a rounded or dome-like non-exophytic lesion and exhibit a benign disease course. Treatment is generally reserved for cosmetic or functional purposes. We present a case of an atypical presentation of an exophytic digital myopericytoma in a 45-year-old female treated with local punch excision.

## Introduction

Myopericytoma is a rare, benign neoplastic growth, first described in 1998 as a tumor of pericytic/myoid origin [1]. Myopericytoma typically affects the soft tissues of the lower extremities, but it has also been shown to affect the head, neck, and upper extremities. The dermis and subcutaneous tissue are usually the affected sites [2]. The cause and pathogenesis are currently not well elucidated. Histologically, myopericytoma is a well-circumscribed nodule with concentric perivascular ovoid or spindle cells containing eosinophilic cytoplasm [3]. As regards immunohistochemistry, all tumors stain at least focally positive for smooth muscle actin (SMA), the majority stain positively for h-Caldesmon, and stain largely negative for desmin, S100, and CD34 [4]. Although myopericytoma is a generally benign growth, recurrence after resection and malignancy have recently been noted [5]. We present a patient with an atypical presentation of a myopericytoma on her left index finger.

## Case presentation

**Figure 1:**
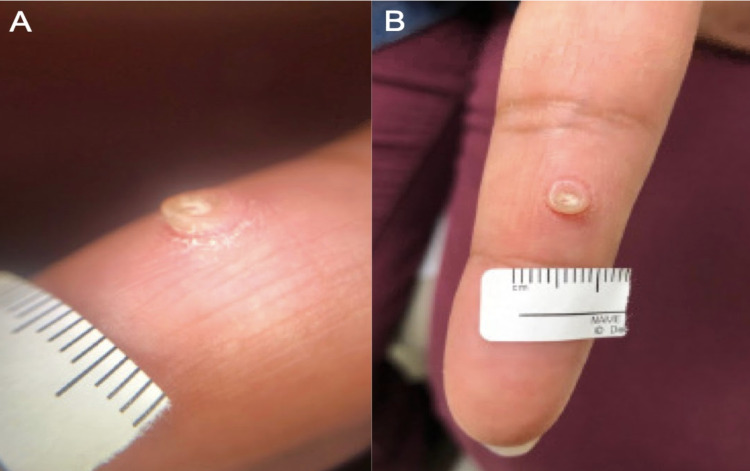
(A) Lateral and (B) volar views of the 5 mm exophytic hyperkeratotic papule.

A 45-year-old female presented with an exophytic growth on her left index finger that was present for several years. She denied any pruritis, pain, or bleeding. The patient tried several over-the-counter wart treatments for the growth with no response. She mentioned that it interfered with her ability to type on a computer keyboard efficiently. On physical examination, a 5 mm exophytic pink papule with a hyperkeratotic rim (Figure 1A, B) was noted on the volar left index finger. Slight erythema directly around the growth was also seen. The clinical differential diagnosis included an acral fibrokeratoma, myofibroma, myrmecia wart, or less likely a glomus tumor. 

**Figure 2:**
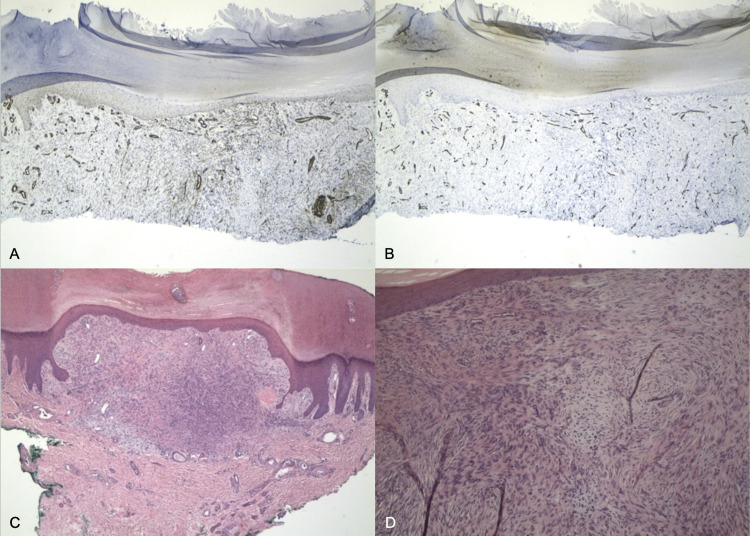
(A,B) Immunohistochemical findings showing SMA focally (+) and CD34 (-), respectively. (C,D) Histopathological findings showing concentric perivascular spindle cells (H&E, original magnifications x40 and x100, respectively).

A punch biopsy of the lesion was performed for histopathological analysis. Immunohistochemical analysis showed that the tumor was focally positive with SMA (Figure 2A) but was negative for CD34 (Figure 2B), epithelial membrane antigen (EMA), and S100. This was most consistent with the diagnosis of myopericytoma. About one month after the initial visit and biopsy, the patient began to notice the formation of a subcutaneous nodule, as well as recurrence of the growth that was initially removed by the punch biopsy. Complete re-excision was performed via a punch biopsy and showed a benign, well-circumscribed, unencapsulated dermal tumor composed of concentric perivascular spindle cells, still consistent with myopericytoma (Figure 2C, D). The margins were free of the tumor cells, and the patient has not had any recurrence at the six-month follow-up.

## Discussion

The clinical presentation described in this case brings to mind several diagnoses, including an acral fibrokeratoma, myofibroma, myopericytoma, myrmecia wart, or less likely a glomus tumor. Often, these entities must be distinguished based on their histopathologic and immunohistochemical features. A myopericytoma is a rare neoplastic growth that can be found anywhere on the body, including the kidneys or brain, but its predilection is more common to the skin of the extremities. Typically, these growths are painless, although painful tumors have been rarely reported [6]. Myopericytoma appears as a well-circumscribed, unencapsulated dermal proliferation of benign ovoid or spindle cells arranged in a concentric perivascular manner [3]. In comparison, a myofibroma lacks the perivascular pattern that is present in myopericytoma and instead has a biphasic zonular pattern [6]. A glomus tumor has nerve involvement, which is absent in myopericytoma. Acral fibrokeratoma has collagen and elastic fiber changes that are not seen in myopericytoma [7,8]. Myopericytoma can be further confirmed with immunohistochemical staining. These tumors are typically positive for SMA and negative for CD34 and S100 [4], as was seen in our case.

The treatment for a myopericytoma is either observation or complete excision [6]. Although myopericytoma is a generally benign tumor with an excellent prognosis, complete removal may be warranted for cosmetic purposes or malignancy potential. Cosmetic factors to consider include the location of the tumor, especially one in a visible part of the body, such as the neck or hand. Another factor is functional obstruction, such as interfering with keyboard typing, as in the case of our patient. Another reason for complete resection is the very rare possibility of malignancy, although only eight such cases have been reported in the literature to date. Surgical excision was noted to be the primary method of successful treatment, with a lack of evidence supporting a chemotherapeutic regimen [9].

## Conclusions

Our patient’s hyperkeratotic and exophytic presentation, as opposed to a more rounded, dome-like, subcutaneous, or non-exophytic lesion seen in the limited number of cases published on cutaneous myopericytoma, made this lesion clinically challenging to diagnose. However, histopathology analysis provided the means to make the diagnosis. This case serves as a reminder to clinicians of the varying clinical characteristics that can be seen in these lesions, in addition to the typical histopathology and immunohistochemical staining patterns. Knowledge of this lesion can lead to more efficient diagnosis and treatment plans.
